# Linking Exercise Intention to Exercise Action: The Moderating Role of Self-Efficacy

**DOI:** 10.3389/fpsyg.2022.921285

**Published:** 2022-07-13

**Authors:** Bin Hou, Linqian Li, Lei Zheng, Yating Qi, Song Zhou

**Affiliations:** ^1^School of Public Administration, Fujian Normal University, Fuzhou, China; ^2^School of Economics and Management, Fuzhou University, Fuzhou, China; ^3^School of Psychology, Fujian Normal University, Fuzhou, China; ^4^Graduate School of Education, University of California, Los Angeles, Los Angeles, CA, United States

**Keywords:** health action process approach, self-efficacy, physical exercise, intention, planning

## Abstract

As physical exercise benefits both physical and psychological health of college students, it is important to promote the habit of physical exercise among them. This study adopted the Health Action Process Approach (HAPA) model to understand the exercise intention–action link and determine the moderating role of self-efficacy. We recruited 242 students from a university in China and asked them to complete a six-wave survey. The survey results indicated that exercise intention was positively related to both coping planning and action planning, which pave the way to performing the action of exercise. However, such mediation effects varied under conditions of self-efficacy. Participants with high self-efficacy exhibited stronger relationships between intention and planning, and between planning and action. The study results suggest that planning has a time-lagged mediation effect in the relationship between intention and action. Additionally, the findings shed light on the moderating role of self-efficacy, which can be useful in developing health-promotion strategies for college students.

## Introduction

Physical exercise refers to any bodily activity that consumes more oxygen than resting (Scully et al., 1998). It benefits both physical and psychological health by reducing the risk of heart disease (Tanaka, 2009; Tarumi and Zhang, 2014) and boosting positive effects (Childs and de Wit, 2014). Indeed, inadequate physical exercise is a risk factor contributing to many chronic diseases (Booth et al., 2012) and other physical health problems (ten Tusscher et al., 2019). According to the World Health Organization (WHO, 2013), lack of physical exercise is strongly related to obesity and overweight, both of which have been linked with diabetes, heart diseases, cancers, and death.

Although it is common knowledge that physical exercise can prevent the development of certain health problems and thus benefit academic performance (Sone et al., 2017; Aro et al., 2018), unfortunately, it seems difficult for most people to convert exercise-related knowledge into actual physical movement (Donnelly et al., 2018). As indicated by Han and Su (2014), the frequency of college students' weekly participation in extracurricular physical activity is low, with only 18.4% of students participating in physical exercise more than four times a week. Besides, the duration of exercise is short, with about 1/3 of students exercising for less than 30 min (32.5%). These results are in line with previous findings. Lack of physical exercise has been observed among college students across countries (Weinstock, 2010; Alves, 2019). In fact, people do not do adequate physical exercise despite having the intention to exercise as they are unable to convert their intentions into actual action (Donnelly et al., 2018). Thus, how to close this intention–action gap and empower individuals to engage in adequate physical exercise has become an important subject of research.

The Health Action Process Approach (HAPA) model provides a powerful description of intention–action linkage. It has been applied to many health-promoting behaviors, including physical exercise (Schwarzer et al., 2007; Scholz et al., 2009; Gaston and Prapavessis, 2014) and healthy diets (Zheng et al., 2021). The HAPA model is regarded as an effective solution for helping convert health-promoting intention into specific action (Schwarzer et al., 2007). This model consists of the following three stages: (1) the pre-intentional stage, which depicts positive internal/external variables that contribute to forming intention; (2) the intentional stage, where individuals develop both action and coping plans based on their goal orientation; and (3) the action stage, where health-promoting behaviors are initiated and maintained. Schwarzer et al. (2007) posited that self-efficacy contributes to the formation of health-promoting plans, as well as the initialization and maintenance of actions (Lippke et al., 2009). The HAPA model has been adopted in the context of many health-promoting behaviors (Chiu et al., 2011; Mak et al., 2015), such as physical activities (Zhou et al., 2021), healthy diets (Hromi-Fiedler et al., 2016), food safety and nutrition (Chow and Mullan, 2010), screening (Paxton, 2016), and safe driving (Dale et al., 2017).

The HAPA model is widely adopted as an approach to promoting the transition from exercise intention to action (Schwarzer and Luszczynska, 2008; Chiu et al., 2011; Maxwell-Smith et al., 2018; Zhang et al., 2019). In this process, planning is considered to be a mediator that closes the gap between exercise intention and actual physical activities (Schwarzer et al., 2007; Barg et al., 2012). As a two-dimensional construct, planning comprises action and coping planning (Schwarzer and Luszczynska, 2008; Pinidiyapathirage et al., 2018). Action planning involves specific details about when, where, and how to implement certain physical exercises, while coping planning specifies the details about how to cope with obstacles in the way of goal achievement. For example, changes in the intention and action planning were found to result in enhanced physical activities among 697 South Korean adults in a longitudinal study (Renner et al., 2007). Importantly, action planning serves as a mediator in the relationship between intention and physical activities (Chiu et al., 2011; Maxwell-Smith et al., 2018). According to Schwarzer and Luszczynska (2008), both action and coping planning play critical roles in the process of turning motivation into action (Fernandez et al., 2016) since the coping planning can help overcome barriers to implementing action plans (Schwarzer et al., 2007). In line with this idea, both action and coping planning have been found to boost physical activities and reduce sedentary behaviors (Fernandez et al., 2016; Sui and Prapavessis, 2018). For example, a recent intervention study showed that college students exhibited reduced sedentary intention and behaviors after they received the action and coping planning intervention (Dillon et al., 2021). It is suggested that both action and coping planning can promote health-related behaviors by enhancing behavioral intention (Smith et al., 2021). In other words, the two factors are regarded as two different but complementary constructs, conducive to the translation of exercise intention into action (Carraro and Gaudreau, 2013; Pinidiyapathirage et al., 2018). Based on the aforementioned arguments, the following hypotheses are proposed:

H1a: Coping planning would mediate the relationship between exercise intention and action.
H1b: Action planning would mediate the relationship between exercise intention and action.

Self-efficacy can enhance the planning function and action formation in the process of transitioning to healthy behaviors (Schwarzer and Luszczynska, 2008; Fernandez et al., 2016). Self-efficacy refers to a belief that one can successfully implement his/her own plans (Schwarzer et al., 2007; Luszczynska et al., 2011). People with strong self-efficacy often exhibit intensified intention toward their goals (Li et al., 2020; Jiatong et al., 2021). It is regarded as an important motivational and volitional factor resulting in health-related behaviors (Schwarzer and Luszczynska, 2008; Fernandez et al., 2016). Given the positive effects of self-efficacy on health-related outcomes, it has been widely considered a focal point of health-promotion approaches (Heuel et al., 2022). Furthermore, self-efficacy can promote the formation of exercise intention and implementation of physical activities (Carraro and Gaudreau, 2013; Pinidiyapathirage et al., 2018). In the HAPA model, three forms of self-efficacy are involved in the pre-intentional and intentional stages as follows (Lippke et al., 2009): action self-efficacy, which contributes to the development of intentions; maintenance self-efficacy; and recovery self-efficacy, which promotes planning.

In this study, general self-efficacy is adopted based on the argument that it can power the transition from intention to action in physical activities. Indeed, self-efficacy is related to incremental planning, with certain consequences for improving physical activities (Parschau et al., 2014; Pinidiyapathirage et al., 2018). As found by Steca et al. (2017) in two follow-up assessments during 6 and 12 months, self-efficacy is associated with increased physical activity among patients. Especially, as pointed out by Luszczynska et al. (2011), action planning is unable to promote physical activity among those without strong self-efficacy. Indeed, people with low self-efficacy often lack clear action plans, thus exhibiting less exercise intention (Feng et al., 2022). Accordingly, high self-efficacy is positively related to planning, which further promotes health-promoting intention. Therefore, self-efficacy seems to boost the intention–action transition by enhancing both action and coping planning.

**Figure 1:**
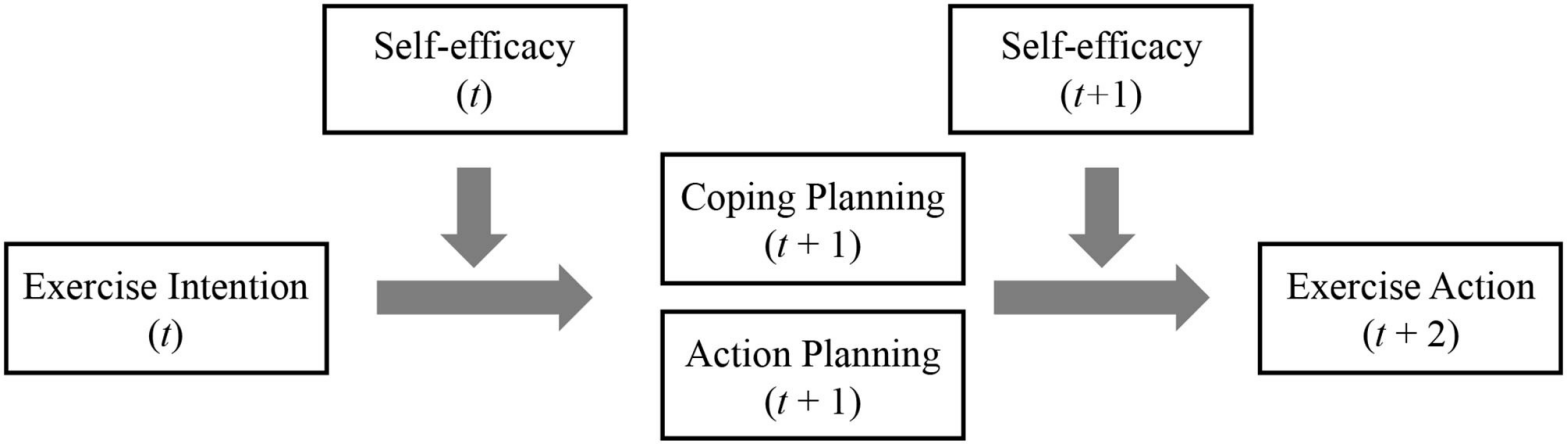
Conceptual model. *t, t*+1, and *t*+2 indicate that the independent variables were measured at time *t*, the mediators were measured at lagged time *t*+1, and the dependent variable measured at lagged time*t*+2.

This study aims to identify whether self-efficacy can serve as a moderator to facilitate the transition from intention to planning, as well as from planning to action (Figure 1). Perhaps, it can moderate the association between intention and the two types of planning, as well as between the two types of planning and action. Accordingly, the following hypotheses are proposed:

H2a: Self-efficacy would moderate the relationship between exercise intention and coping planning. The association between them is stronger in people with high self-efficacy than in those with low self-efficacy.
H2b: Self-efficacy would moderate the relationship between exercise intention and action planning. The association between them is stronger in people with high self-efficacy than in those with low self-efficacy.

## Methods

### Participants and Research Design

We conducted a sample size estimation for statistical hypothesis testing. The effect sizes were extracted from previous studies that examined the mediation effect of planning in the relationship between intention and action (Zhou et al., 2016; Zhang et al., 2019; Zheng et al., 2021). The results showed that the minimum required sample size was 215 when the statistical power (1 – β) was set as 0.90 or above. We recruited more than our planned participants because some participants might dropout in this six-wave survey at a 2-week interval. A total of 242 participants were recruited by advertisements in several optional courses. There was a link presented in each advertisement, which redirected them to an online survey solution platform (http://www.wjx.com). All participants were informed to read the online consent, which was approved by the Ethics Committee of Fujian Normal University. Only those who accepted the consent could continue the study. They were required to complete the six-wave survey during the day, which included exercise intention, coping planning, action planning, self-efficacy, and exercise action in each wave.

There were 218 university students who volunteered and finished all six-wave surveys. According to their demographic information, all of them were health undergraduate students (mean age = 19.53 ± 1.01; 64 men and 154 women). They also reported their family socioeconomic status (SES) by the social ladder ranging from the bottom (1) to the top (10) of the social scale (Ghaed and Gallo, 2007). Their mean family SES was 4.74 (SD = 1.83).

### Measures

The physical exercise intention, coping planning, action planning, and self-efficacy were adopted from the Chinese version of the HAPA model (Schwarzer et al., 2007), and the exercise action was adopted from the Chinese version of physical activity scale (Renner and Schwarzer, 2005).

Physical exercise intention was measured by three items (“I intend to practice physical exercise on a regular basis,” “I intend to practice exercise for at least 30 min every occasion,” and “I intend to practice physical exercise several times every week”). Participants rated on a 5-point scale (1 = completely disagree, 2 = partially disagree, 3 = neither agree nor disagree, 4 = partially agree, 5 = completely agree). The Cronbach's alpha values were 0.92–0.95 in this study.

Coping planning and action planning each include three items. The statements for coping planning were “I have already planned what to do if something interferes with my plans,” “I have already planned what to do in difficult situations to stick to my intentions,” and “I have already planned what to do with possible setbacks.” The statements for coping planning were “I have already planned on which days I will do exercise,” “I have already planned where to practice exercise,” and “I have already planned how to practice exercise (what kind of exercise will I practice).” Participants rated on a 5-point scale (1 = not at all true, 2 = slightly true, 3 = moderately true, 4 = quite true, and 5 = completely true). The Cronbach's alpha values were 0.92–0.97 for coping planning and 0.94–0.96 for action planning in this study.

Self-efficacy scale includes three items (i.e., “I am confident that I can practice physical exercise on a regular basis,” “I am confident I can practice physical exercise at least once a week,” and “I believe I can practice exercise for at least 90 min a week”). Participants rated on a 5-point scale (1 = not true at all, 2 = not true, 3 = unsure, 4 = true, and 5 = exactly true). The Cronbach's alpha values were 0.83–0.90 in this study.

Exercise action was measured by a single item: “Have you been exercising on a regular basis during the last two weeks?” Participants rated on a 4-point scale (1 = no, 2 = yes, with longer interruptions, 3 = yes, with short interruptions, and 4 = yes, without interruption).

### Data Analyzes

All data analyses were conducted using R 4.0.2. First, descriptive analysis and correlation analysis were conducted. Second, we conducted an attrition analysis to examine the mean differences for all variables between follow-up and dropout data. Third, the exploratory factor analysis was conducted to assess the possible bias caused by the common method using psych package in R. Finally, we conducted multilevel models using lme4 package in R. We included the physical exercise intention, coping planning, action planning, exercise action as within-level variables, and demographic variables as between-level variables. The mediation effects were tested by the Monte Carlo simulation approach.

## Results

### Descriptive Statistics

The descriptive information of all variables, including means, standard deviations, and Pearson's correlation coefficients, are presented in Table 1.

**Table 1:** Means, standard deviations, and Pearson's correlation coefficients for all variables.

	**Mean**	**SD**	**1**	**2**	**3**	**4**	**5**	**6**	**7**	**8**	**9**
1. Exercise Intention*^*t*^*	3.83	0.95	0.68								
2. Coping Planning*^*t*^*	3.39	1.07	0.69**	0.67							
3. Coping Planning*^*t*+1^*	3.37	1.07	0.65**	0.74**	0.69						
4. Action Planning*^*t*^*	3.61	1.06	0.73**	0.81**	0.66**	0.69					
5. Action Planning*^*t*+1^*	3.60	1.06	0.62**	0.67**	0.81**	0.76**	0.67				
6. Self-Efficacy*^*t*^*	3.72	0.94	0.83**	0.76**	0.67**	0.81**	0.68**	0.70			
7. Self-Efficacy*^*t*+1^*	3.68	0.95	0.70**	0.66**	0.77**	0.69**	0.80**	0.78**	0.69		
8. Exercise Action*^*t*+1^*	2.04	0.86	0.42**	0.46**	0.50**	0.46**	0.50**	0.48**	0.51**	0.55	
9. Exercise Action*^*t*+2^*	2.05	0.86	0.41**	0.44**	0.46**	0.44**	0.46**	0.43**	0.48**	0.62**	0.58

^**^p < 0.01; t, t+1, and t+2 indicate that the independent variables were measured at time t, the mediators were measured at lagged time t+1, and the dependent variable measured at lagged time t+2; the diagonal values were intraclass correlation coefficients.

### Attrition Analysis and Common Method Variance

In attrition analysis, the results showed that there were nonsignificant differences for exercise intention (*t* = 1.34, *p* = 0.182), coping planning (*t* = 1.08, *p* = 0.279), action planning (*t* = 0.42, *p* = 0.672), self-efficacy (*t* = 0.56, *p* = 0.576), and exercise action (*t* = 0.75, *p* = 0.455).

We employed Harman's one-factor test to estimate the percentage of the common method variance. The results showed that the first factor accounted for 26% of the total variance, which satisfied the criterion (50%) (Fuller et al., 2016).

### The Time-Lagged Mediation Effects of Coping Planning and Action Planning

We examined the mediation effects of coping planning and action planning in the relationship between physical exercise intention and action. The data structure was reorganized to examine the time-lagged effects of coping planning and action planning. Thus, the exercise intention was measured at T1, the coping planning and action planning were measured at T2 (i.e., 2 weeks later), and physical exercise action was measured at T3 (i.e., 4 weeks later). As the measurements were nested within participants, the multilevel models were developed. The null model showed that all intraclass correlation coefficients were acceptable (0.55–0.70; see Table 1) for multilevel modeling.

As shown in Table 2, this study found that physical exercise intention at T1 was positively related to coping planning (T2; β = 0.29, SE = 0.03, *p* < 0.001) and action planning (T2; β = 0.16, SE = 0.03, *p* < 0.001), after controlling demographic variables and baseline levels. Moreover, considering predictors of the physical exercise action at T3, the significant correlates included coping planning (T2; β = 0.11, SE = 0.04, *p* = 0.004) and action planning (T2; β = 0.09, SE = 0.04, *p* = 0.016).

**Table 2:** The results of multilevel regression.

	**Model 1** **Coping planning*^***t*+1**^*** **β (s.e.)**	**Model 2** **Action planning*^***t*+1**^*** **β (s.e.)**	**Model 3** **Exercise action*^***t*+1**^*** **β (s.e.)**
**Between-Level**			
Intercept	3.38(0.02)***	3.59(0.02)***	2.04(0.03)***
Women	−0.08(0.02)*	−0.02(0.02)	−0.07(0.03)*
Age	−0.03(0.02)	0.01(0.02)	−0.01(0.03)
Family SES	0.02(0.02)	0.02(0.02)	0.02(0.03)
**Within-Level**			
Exercise action*^*t*^*			0.28(0.03)***
Coping planning*^*t*^*	0.53(0.03)***		0.11(0.04)**
Action planning*^*t*^*		0.68(0.03)***	0.09(0.04)*
Exercise intention*^*t*^*	0.29(0.03)***	0.16(0.03)***	
Within-Level *R^2^*	0.51	0.59	0.30

^*^p < 0.05, ^**^p < 0.01, ^***^p < 0.001; t and t+1 indicate that the independent variables were measured at time t, and the dependent variables were measured at lagged time t+1; Family SES, family socioeconomic status.

**Figure 2:**
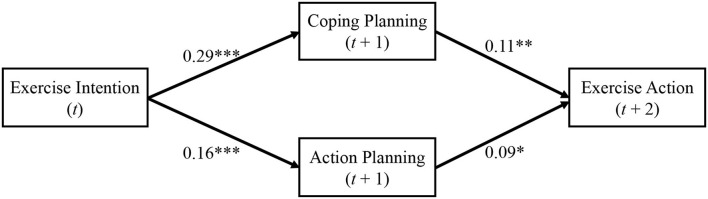
The results of mediation model. * *p* < 0.05, ** *p* < 0.01, *** *p* < 0.001; *t, t*+1, and *t*+2 indicate that the independent variables were measured at time *t*, the mediators were measured at lagged time *t*+1, and the dependent variable measured at lagged time *t*+2.

Additionally, the results indicated a significant mediating effect of coping planning and action planning at T2 in the associations between that physical exercise intention at T1 and action at T3 (coping planning: effect size = 0.03, SE = 0.01, 95% CI [0.01, 0.06]; action planning: effect size = 0.02, SE = 0.01, 95% CI [0.002, 0.03]; Figure 2).

### The Moderation Effects of Self-Efficacy

**Figure 3:**
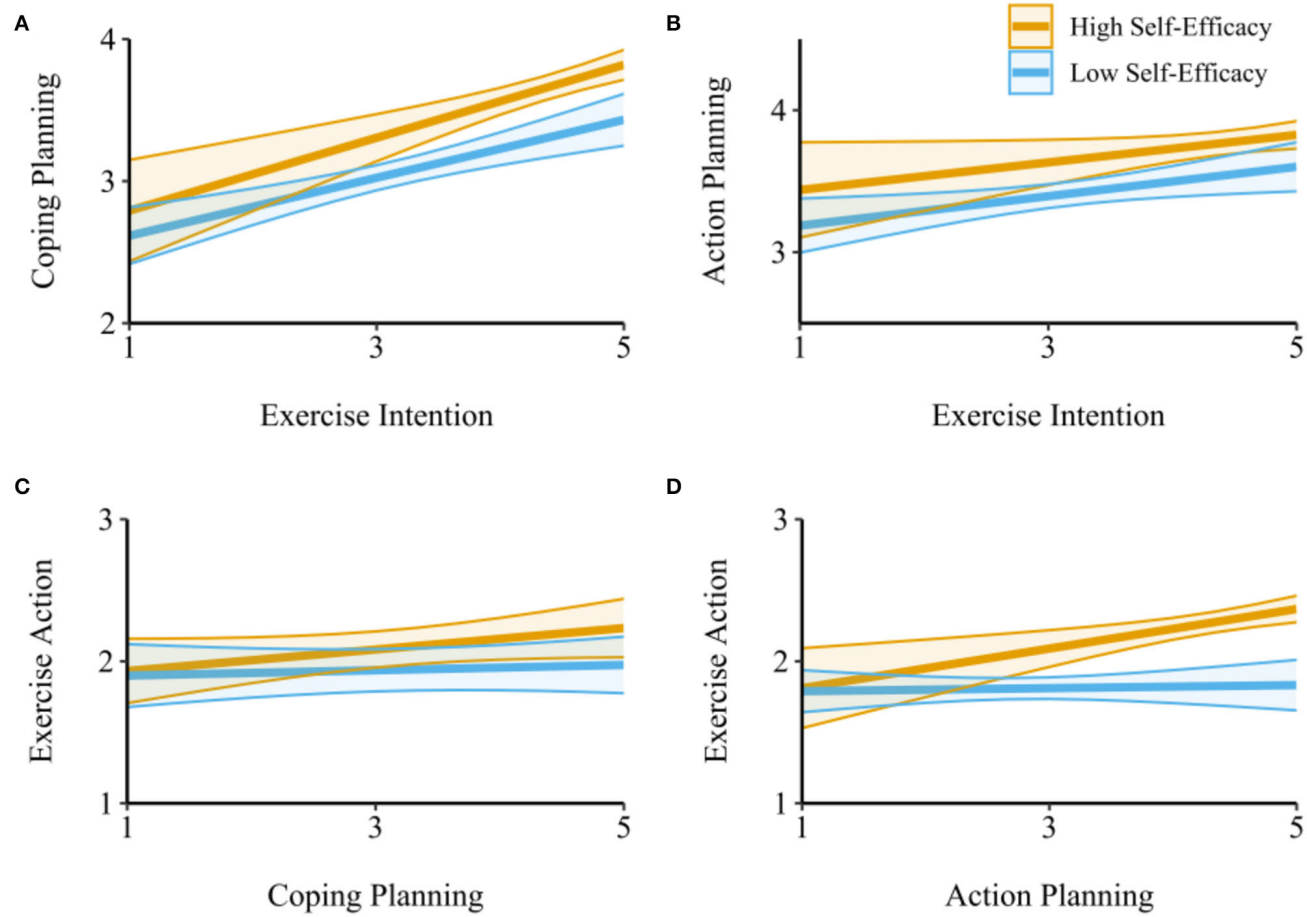
The results of simple slope analysis. The moderating effect of self-efficacy on the relationship between exercise intention and coping planning **(A)**, between exercise intention and action planning **(B)**, between coping planning and exercise action **(C)**, and between action planning and exercise action **(D)**.

Next, we tested the moderating role of self-efficacy in the relationships between exercise intention and coping/action planning, and between coping/action planning and exercise action. As shown in Table 3, the self-efficacy at T1 moderated the relationship between physical exercise intention (T1) and coping planning (T2; β = 0.06, SE = 0.02, *p* = 0.015; Figure 3A), but not between physical exercise intention (T1) and action planning (T2; β = 0.01, SE = 0.02, *p* = 0.841; Figure 3B). In particular, the association between physical exercise intention and coping planning was stronger among people with high self-efficacy (β = 0.29, SE = 0.06, *p* < 0.001) compared to those with low self-efficacy (β = 0.18, SE = 0.05, *p* < 0.001).

**Table 3:** The results of multilevel moderation regression.

	**Model 4** **Coping planning*^***t*+1**^*** **β (s.e.)**	**Model 5** **Action planning*^***t*+1**^*** **β (s.e.)**	**Model 6** **Exercise action*^***t*+1**^*** **β (s.e.)**
**Between-Level**			
Intercept	3.33(0.03)***	3.59(0.02)***	1.98(0.03)***
Women	−0.06(0.03)*	−0.02(0.02)	−0.05(0.03)
Age	−0.05(0.02)	0.01(0.02)	−0.01(0.03)
Family SES	0.02(0.03)	0.02(0.02)	0.01(0.03)
Within-Level			
Exercise action*^*t*^*			0.29(0.03)***
Coping planning*^*t*^*	0.42(0.04)***		0.05(0.04)
Action planning*^*t*^*		0.63(0.04)***	0.05(0.04)
Exercise intention*^*t*^*	0.23(0.05)***	0.1(0.04)*	
Self-Efficacy*^*t*^*	0.16(0.04)***	0.12(0.05)*	
EI*^*t*^* x SE*^*t*^*	0.06(0.02)*	0.01(0.02)	0.17(0.04)***
CP*^*t*^* x SE*^*t*^*			−0.03(0.03)
AP*^*t*^* x SE*^*t*^*			0.09(0.03)**
Within-Level *R^2^*	0.53	0.59	0.34

^*^p < 0.05, ^**^p < 0.01, ^***^p < 0.001; t and t+1 indicate that the independent variables were measured at time t, and the dependent variables were measured at lagged time t+1; family SES, family socioeconomic status; EI ^t^ × SE^t^ is the interaction of exercise intention and self-efficacy; CP ^t^ × SE ^t^ is the interaction of coping planning and self-efficacy; AP ^t^ × SE ^t^ is the interaction of action planning and self-efficacy.

Similarly, the self-efficacy at T2 moderated the relationship between action planning (T2) and physical exercise action (T3; β = 0.09, SE = 0.03, *p* = 0.006), but not between coping planning (T2) and physical exercise intention (T3; β = −0.03, SE = 0.03, *p* = 0.380; Figure 3C). In particular, the association between action planning and exercise action was stronger among people with high self-efficacy (β = 0.15, SE = 0.04, *p* < 0.001) compared to those with low self-efficacy (β = 0.01, SE = 0.04, *p* = 0.779; Figure 3D).

**Figure 4:**
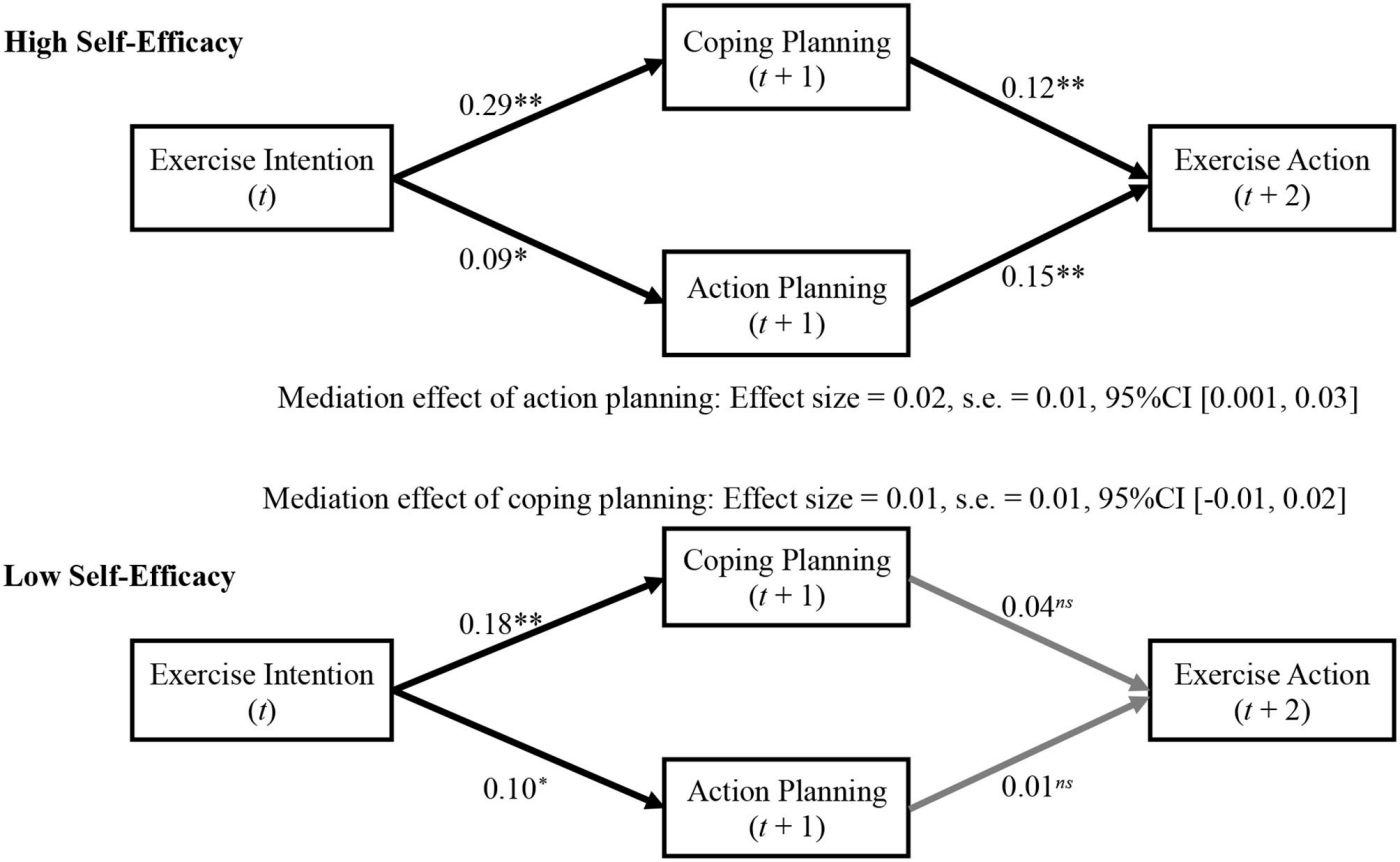
The results of moderated mediation model. Note: * *p* < 0.05, ** *p* < 0.01; *t, t*+1, and *t*+2 indicate that the independent variables were measured at time *t*, the mediators were measured at lagged time *t*+1, and the dependent variable measured at lagged time *t*+2.

We further examined the moderation effects of self-efficacy on the mediation path from physical exercise intention to exercise action. The results showed that, for people with high self-efficacy, both coping planning and action planning served as mediators in the relationship between exercise intention and exercise action (coping planning: effect size = 0.04, SE = 0.01, 95% CI [0.01, 0.06]; action planning: effect size = 0.02, SE = 0.01, 95% CI [0.001, 0.03]). However, neither those mediation effects were nonsignificant for people with low self-efficacy (coping planning: effect size = 0.01, SE = 0.01, 95% CI [−0.01, 0.02]; action planning: effect size = 0.001, SE = 0.01, 95% CI [−0.01, 0.01]; Figure 4).

## Discussion

This study confirmed that action and coping planning had time-lagged mediation effects on the relationship between intention and action for physical exercises. Moreover, self-efficacy was found to be a moderator that promoted both intention–planning transition and planning–action for physical exercise. These results point to a moderated mediation effect of self-efficacy on the transition from intention to action in the context of physical exercise.

### Time-Lagged Mediation Effects of Planning

This study showed that both action and coping planning serve as parallel mediators in the relationship between intention and action in the context of physical exercise. It suggested that action and coping planning are two different but complementary factors that facilitate the transition from intention to action. Action planning translates the intention into specific details and actions, such as when, where, and how to exercise, while coping planning consists of preparing for possible obstacles ahead. Importantly, coping planning is found to promote the transition from action planning to action as it prepares the individual to overcome possible obstacles in the future (Schwarzer et al., 2007).

The results of this study provided empirical evidence for the HAPA model in explaining physical activities among Chinese college students. In line with the HAPA model (Schwarzer and Luszczynska, 2008; Fernandez et al., 2016), physical activity intention is positively related to both action and coping planning, and then further related to action. For Chinese college students, performing physical exercise can boost their physical and psychological health. As indicated in previous literature, planning is a critical aspect of intention–action transition in promoting physical exercise (Schwarzer et al., 2007; Barg et al., 2012). Thus, further studies should consider planning management as an approach to promote intention–action transition to enhance physical exercise.

A physical activity guideline developed by WHO (2013) recommended that adults must engage in at least 150-min moderate-to-vigorous physical exercise per week. As proposed by previous research, low-to-moderate physical exercises are most optimal for improving physical health and cognitive function (Kashihara et al., 2019). Although vigorous physical exercise has been thought to result in fatigue, Szymczak et al. (2020) posit that an increase in vigorous physical exercise leads to a stronger sense of behavioral change than low-to-moderate physical exercise. It seems that the physical and psychological functions of physical exercises vary under different exercise intensities. Future research must examine the time-lagged mediation effects of planning in all conditions, including low, moderate, and vigorous physical exercises.

### The Moderating Effects of Self-Efficacy on Physical Exercise

This study identified the moderating role of self-efficacy in the relationships between intention and coping/action planning for exercises, as well as between coping/action planning and exercise action. This finding demonstrates that self-efficacy can enhance the transition from intention to action for physical exercise. This is partly consistent with the result of previous studies: self-efficacy leads to enhanced intentions and planning for exercises (Scholz et al., 2009; Ochsner et al., 2013).

This study found that self-efficacy serves as a moderator, providing new empirical evidence for the role of self-efficacy in the HAPA model. According to previous research, self-efficacy contributes to forming intentions, making plans, and improving action in the process of healthy behavioral changes (Schwarzer and Luszczynska, 2008; Parschau et al., 2014; Fernandez et al., 2016; Pinidiyapathirage et al., 2018). This study confirms that self-efficacy can enhance the intention–action linkage while highlighting the positive function of self-efficacy in cultivating health-promoting behaviors.

This study merely focused on general self-efficacy. However, there are three forms of self-efficacy in the HAPA model (Lippke et al., 2009), which serve different functions across different stages in the model (Barg et al., 2012; Parschau et al., 2014). We suggest future studies to further explore the moderating functions of the three types of self-efficacy in different stages of the health-promotion model.

## Implications

Physical exercise is generally regarded as an effective approach to alleviating academic stress among college students (Li et al., 2021; Zhou et al., 2021). Hence, it is important to promote the transition from exercise intention to practical action among students. Planning management can help college students formulate their exercise plans and prepare for possible obstacles in the way of their goals. The findings of this study can help plan training strategies for college students and enable them to implement their physical exercise plans, provided they have strong exercise intentions. An important finding of this study is that self-efficacy moderates the intention–action relationship. Given that the intention–action transition in terms of exercises is stronger for college students with high self-efficacy than those with low self-efficacy, specific strategies should be developed for them. First, schools and teachers must encourage students to commit to improving their health by developing specific and detailed institutional health-promotion plans and implementation documents. Second, schools should actively organize meaningful physical health activities to create a favorable sports environment on campus. Teachers should offer timely encouragement to students to enhance their confidence and arouse interest in physical education, thus effectively fortifying their willingness to participate in physical activities and ultimately enhancing their self-efficacy. Third, parents of such students should encourage them to deliver positive changes in their physical exercise intention while monitoring the implementation of their physical exercise plans in a timely manner.

## Limitation and Future Direction

There are some limitations to this study. First, although it consisted of six rounds of longitudinal surveys with 2-week intervals between them, the measurements are merely based on self-report scales. This made it impossible to infer causal relationships. Hence, it is suggested that further studies be conducted employing other research designs for similar surveys, such as field studies. Second, the intention for physical exercise is measured with a single item, thus limiting the reliability of physical exercise metrics, because no reliability information can be obtained. Future studies are encouraged to adopt portable tools to record data on physical exercise under different exercise intensities. Third, the participants of this study are all Chinese college students recruited from mainland China; hence, caution is needed in generalizing these findings to other populations or countries.

## Conclusion

In this study, both action planning and coping planning acted as time-lagged mediators in the relationship between the intention of exercising and the actual action of exercising. It provided empirical evidence for the HAPA model of physical exercise. Moreover, this study found that self-efficacy served as a moderator that helped individuals transition from intention to planning, as well as from planning to action in the context of physical exercise. The findings suggest that further studies develop strategies to promote self-efficacy for promoting transition from exercise intention to exercise action.

## Data Availability Statement

The raw data supporting the conclusions of this article will be made available by the authors, without undue reservation.

## Ethics Statement

The studies involving human participants were reviewed and approved by the Ethics Committee of Fujian Normal University. The patients/participants provided their written informed consent to participate in this study.

## Author Contributions

BH was responsible for writing. LZ contributed to the conception of the work and drafting the article. LZ, SZ, and LL was responsible for data analysis and interpretation. YQ and BH contributed to revising the article for important intellectual content. SZ and LL contributed to the data collection and gave important revising suggestions. All authors contributed to the article and approved the submitted version.

## Funding

This study was supported by the National Social Science Foundation of China (20CSH073) and the Fujian Social Science Foundation for Education Product (JAS19029) awarded to LZ and the Key Project of Fuzhou Social Science Planning (2021FZB08) and the Education and Scientific Research Project of Young and Middle-aged Teachers of Fujian Provincial Education Department (Social Science) (JAS19035) awarded to BH.

## Conflict of Interest

The authors declare that the research was conducted in the absence of any commercial or financial relationships that could be construed as a potential conflict of interest.

## Publisher's Note

All claims expressed in this article are solely those of the authors and do not necessarily represent those of their affiliated organizations, or those of the publisher, the editors and the reviewers. Any product that may be evaluated in this article, or claim that may be made by its manufacturer, is not guaranteed or endorsed by the publisher.
